# Gypenosides improve diabetic cardiomyopathy by inhibiting ROS‐mediated NLRP3 inflammasome activation

**DOI:** 10.1111/jcmm.13743

**Published:** 2018-07-11

**Authors:** Hailong Zhang, Xi Chen, Beibei Zong, Hongmin Yuan, Zhizeng Wang, Yinxiang Wei, Xuance Wang, Guangchao Liu, Jun Zhang, Shulian Li, Guanchang Cheng, Yaohui Wang, Yuanfang Ma

**Affiliations:** ^1^ Joint National Laboratory for Antibody Drug Engineering Key Laboratory of Cellular and Molecular Immunology of Henan Province School of Basic Medicine Henan University Kaifeng China; ^2^ Department of General Pathology Huaihe Hospital Henan University Kaifeng China; ^3^ Department of Thyroid Breast Surgery Huaihe Hospital Henan University Kaifeng China; ^4^ Centre for Translational Medicine Huaihe Hospital Henan University Kaifeng China; ^5^ Department of Cardiac Surgery Huaihe Hospital Henan University Kaifeng China

**Keywords:** cytochrome *c*, diabetic cardiomyopathy, gypenosides, high glucose, NLRP3 inflammasome, ROS

## Abstract

NLRP3 inflammasome activation plays an important role in diabetic cardiomyopathy (DCM), which may relate to excessive production of reactive oxygen species (ROS). *Gypenosides* (*Gps*), the major ingredients of *Gynostemma pentaphylla* (*Thunb*.) *Makino*, have exerted the properties of anti‐hyperglycaemia and anti‐inflammation, but whether *Gps* improve myocardial damage and the mechanism remains unclear. Here, we found that high glucose (HG) induced myocardial damage by activating the NLRP3 inflammasome and then promoting IL‐1β and IL‐18 secretion in H9C2 cells and NRVMs. Meanwhile, HG elevated the production of ROS, which was vital to NLRP3 inflammasome activation. Moreover, the ROS activated the NLRP3 inflammasome mainly by cytochrome *c* influx into the cytoplasm and binding to NLRP3. Inhibition of ROS and cytochrome *c* dramatically down‐regulated NLRP3 inflammasome activation and improved the cardiomyocyte damage induced by HG, which was also detected in cells treated by *Gps*. Furthermore, *Gps* also reduced the levels of the C‐reactive proteins (CRPs), IL‐1β and IL‐18, inhibited NLRP3 inflammasome activation and consequently improved myocardial damage in vivo. These findings provide a mechanism that ROS induced by HG activates the NLRP3 inflammasome by cytochrome *c* binding to NLRP3 and that *Gps* may be potential and effective drugs for DCM via the inhibition of ROS‐mediated NLRP3 inflammasome activation.

## 1 INTRODUCTION

Diabetic cardiomyopathy (DCM) is the primary cause of overt heart failure and death in diabetic individuals and affects nearly 12% of diabetic patients.^1^, ^2^, ^3^ Ma et al recently report that the heart failure rate in patients with diabetes is four times that in people without diabetes.^4^ Strategies regulating blood glucose cannot effectively inhibit the incidence of DCM and improve cardiac dysfunction.^5^ Moreover, lowering glucose levels could induce adverse cardiovascular outcomes.^6^ Thus, it is important to seek out the molecular mechanisms that control the progression of DCM and to develop novel methods.

Inflammasomes are cytosolic, multiprotein complexes that are composed of a NOD‐like receptor (NLR) or the absent in melanoma (AIM2)‐like receptors, ASC and pro‐caspase‐1.^7^, ^8^, ^9^ Among the NLRs, NLRP3 is best known for sensing the widest array of stimuli, such as nigericin, ATP, ROS, MSU and cholesterol.^10^, ^11^, ^12^ When the host receives a signal stimulus, NLRP3 itself can undergo oligomerization, increasing pro‐caspase‐1 through the adapter protein ASC, which activates caspase‐1 and then leads to the cleavage of the interleukin‐1β precursor (pro‐interleukin‐1β) and the interleukin‐18 precursor (pro‐interleukin‐18), causing corresponding inflammation factor IL‐1β and IL‐18 generation, thus inducing inflammation and necrosis (pyroptosis) and participating in the inflammatory response of cell and tissue.^7^, ^13^, ^14^

Although the inflammasome plays an important role in the innate immune response and anti‐infection immune responses, inflammasome overactivation was shown to be involved in some chronic inflammatory diseases, such as Alzheimer's, ulcerative colitis, atherosclerosis and type 2 diabetes and its complications.^15^, ^16^, ^17^, ^18^, ^19^ Hence, inhibiting inflammasome activation may be a promising strategy for DCM. In rats with type 2 diabetes, the NLRP3 inflammasome is up‐regulated and promotes the development of DCM.^20^, ^21^, ^22^ Inhibiting the activation of the NLRP3 inflammasome can suppress the release of proinflammatory cytokines and attenuate the development of DCM.^23^, ^24^ In addition, NLRP3 gene silencing alleviates cardiac inflammation and exerts a protective effect on DCM.^25^ However, the molecular mechanisms by which high glucose (HG) activates the NLRP3 inflammasome in cardiomyocytes remain to be further explored.

As a traditional Chinese medicine, *Gynostemma pentaphylla* (*Thunb*.) *Makino* exerts anti‐hypertensive, anti‐ageing, anti‐hyperlipidemia, anti‐hyperglycaemia and anti‐inflammation effects.^26^, ^27^, ^28^, ^29^, ^30^ As a result, *Gynostemma* has been used to improve symptoms of several diseases for centuries, including hyperlipidemia, hepatitis, atherosis and diabetes. *Gypenosides* (*Gps*), the major ingredient of *Gynostemma*, also possess anti‐inflammatory, cardioprotective and neuroprotective properties. In astrocytes and mice stimulated by LPS, *Gps* suppress NF‐κB activation and reduce IL‐1β secretion.^29^ Thus, *Gps* may inhibit NLRP3 inflammasome activation and improve DCM.

In this study, we utilized HG‐stimulated H9C2 and high‐fat‐diet/STZ‐fed rats to study the mechanisms of NLRP3 inflammasome activation. Our study identifies that HG induces mitochondria damage and results in the overproduction of reactive oxygen species (ROS) and the release of cytochrome *c*, which lead to NLRP3 inflammasome activation and cell death. We further find that ROS activates the NLRP3 inflammasome, which is dependent on cytochrome *c* in H9C2 treated with HG. Besides these, HG‐induced NLRP3 inflammasome activation is inhibited by *Gps*, which reduces IL‐1β release and improves DCM. Consequently, *Gps* may be a potential therapeutic strategy for DCM.

## 2 METHODS

### 2.1 Cell culture and treatment

H9C2 cells were purchased from the Library of Typical Culture of the Chinese Academy of Sciences (Shanghai, China). The cells were cultured in a DMEM medium supplemented with D‐glucose (5.5 mmol/L), 10% FBS, penicillin (100 U/mL) and streptomycin (100 mg/mL). Moreover, neonatal rat ventricular myocytes (NRVMs) were obtained and cultured as previously described.^31^ In HG‐cultured groups, the medium contained 25 or 35 mmol/L of D‐glucose. Z‐YYAD‐FMK, NAC, and cyclosporin A were obtained from Abcam Trading (Shanghai) Company Ltd. *Gps* were purchased from Yuanye Biology Company (Shanghai) and dissolved in PBS for in vitro and in vivo experiments.

### 2.2 Rats

SD rats (7 weeks of age) were purchased from Beijing Vital River Laboratory Animal Technology Co. Ltd (Beijing, China). All animal studies and experiments were permitted by the Animal Experimental Ethics Committee of Henan University. Male rats were bred in a specific pathogen‐free facility. After being fed a basal diet for 1 week, rats were randomly divided into chow diet (n = 6) and high‐fat diet (n = 30) groups. Four weeks later, 35 mg/kg of streptozotocin (STZ, Solarbio, China) was administrated intraperitoneally. One week later, rats fasted for 10 hours and then blood glucose levels were measured by a glucometer (Roche, Germany). The rats whose blood glucose exceeded 12 mmol/L had diabetes and were used for the following study. Then, the diabetic rats were respectively treated with Z‐YVAD‐FMK (caspase‐1 inhibitor, intravenous, 60 mg/kg, n = 6), *Gps* (intragastric, 200 mg/kg, n = 6), or vehicle (PBS, n = 6) once a day for 8 weeks. Once a week, blood glucose and body weight were detected and recorded. Finally, all rats were killed under anaesthesia. Blood was collected from the carotid artery and centrifuged for 10 minutes at 1800 ***g*** at 4°C to obtain the serum. The hearts were rapidly frozen in liquid nitrogen to extract the related genes and proteins or embedded in 4% paraformaldehyde for pathological analysis after lavage.

### 2.3 Cell vitality and cell apoptosis

H9C2 cells and NRVMs were maintained with different concentrations of glucose (5.5, 25 and 35 mmol/L), along with Z‐YYAD‐FMK, NAC, cyclosporin A or *Gps*. Several hours later (6, 12, 24, 36 and 48 hours), the supernatant of H9C2 cells was obtained to detect cell vitality using LDH. MTS, TUNEL and FCM were used to analyse cell vitality and cell apoptosis, which have been described in previous publications.^25^, ^29^, ^32^, ^33^

### 2.4 Western blot, reverse‐transcription and real‐time PCR

Western blot (WB) and real‐time PCR **(**RT‐PCR) were carried out as previously described.^34^ Antibodies for NLRP3 and ASC were purchased from Santa Cruz Technology (Santa Cruz, CA). Antibodies for caspase‐1, IL‐1β, cytochrome *c*, VDAC, β‐actin, GAPDH and the secondary HRP‐conjugated antibody were obtained from Cell Signaling Technology (CST). Primers for ASC, NLRP3, caspase‐1 and IL‐1β genes were obtained from Sangon Biotech Company (Shanghai). The primer sequences are presented in Table S1.

### 2.5 Immunofluorescence

Immunofluorescence was used to detect the expression of NLRP3 inflammasome markers as previously described.^35^ The first antibodies contained anti‐NLRP3, ASC, caspase‐1 and IL‐1β antibodies. An Alexa Fluor 488 goat anti‐rabbit IgG (H+L) antibody and an Alexa Fluor 594 goat antimouse IgG (H+L) antibody were used as secondary antibodies. Fluorescence was detected with an inverse fluorescent microscope (Nikon, Japan).

### 2.6 ROS levels

Reactive oxygen species levels were detected with a peroxide‐sensitive fluorescent probe 2′, 7′‐dichlorofluorescein diacetate (DCFH‐DA). After being treated with HG and/or NAC or *Gps*, H9C2 cells were washed with PBS three times and incubated with DCFH‐DA for 30 minutes away from light. The fluorescence was detected with a flow cytometer (BD, America).

### 2.7 ELISA

Supernatants of the cell culture or serum were used to measure IL‐1β, IL‐18 and C‐reactive protein (CRP) (Thermo Fisher Scientific) according to the manufacturer's instructions.

### 2.8 Histological examination

After the rats were killed, their hearts were perfused with PBS and bisected at the mid‐ventricular level. Then, the hearts were fixed in 4% paraformaldehyde and embedded in paraffin. Four micrometre sections were stained with haematoxylin and eosin (H&E), and images were randomly captured by microscope.

### 2.9 Co‐immunoprecipitation

Co‐immunoprecipitation (CoIP) was carried out as previously described.^36^ 1 mg of cell lysate was treated with protein A/G agarose beads for 1 hour. After being centrifuged for 10 minutes, the supernatants were incubated with an anti‐NLRP3 antibody overnight at 4°C. Then, 100 μL protein A/G agarose beads were added to the supernatants and mixed for 2 hours. The beads were centrifuged and washed five times with a lysis buffer. The samples were subjected to immunoblotting with the anti‐ cytochrome *c* antibody and corresponding secondary antibodies.

### 2.10 Statistical analysis

All data were shown as mean ± SD. Samples were analysed using a one‐way ANOVA on SPSS 16.0 software. *P* < .05 was considered a statistically significant difference.

## 3 RESULTS

### 3.1 HG‐induced cell injury in cardiomyocytes

**Figure 1:**
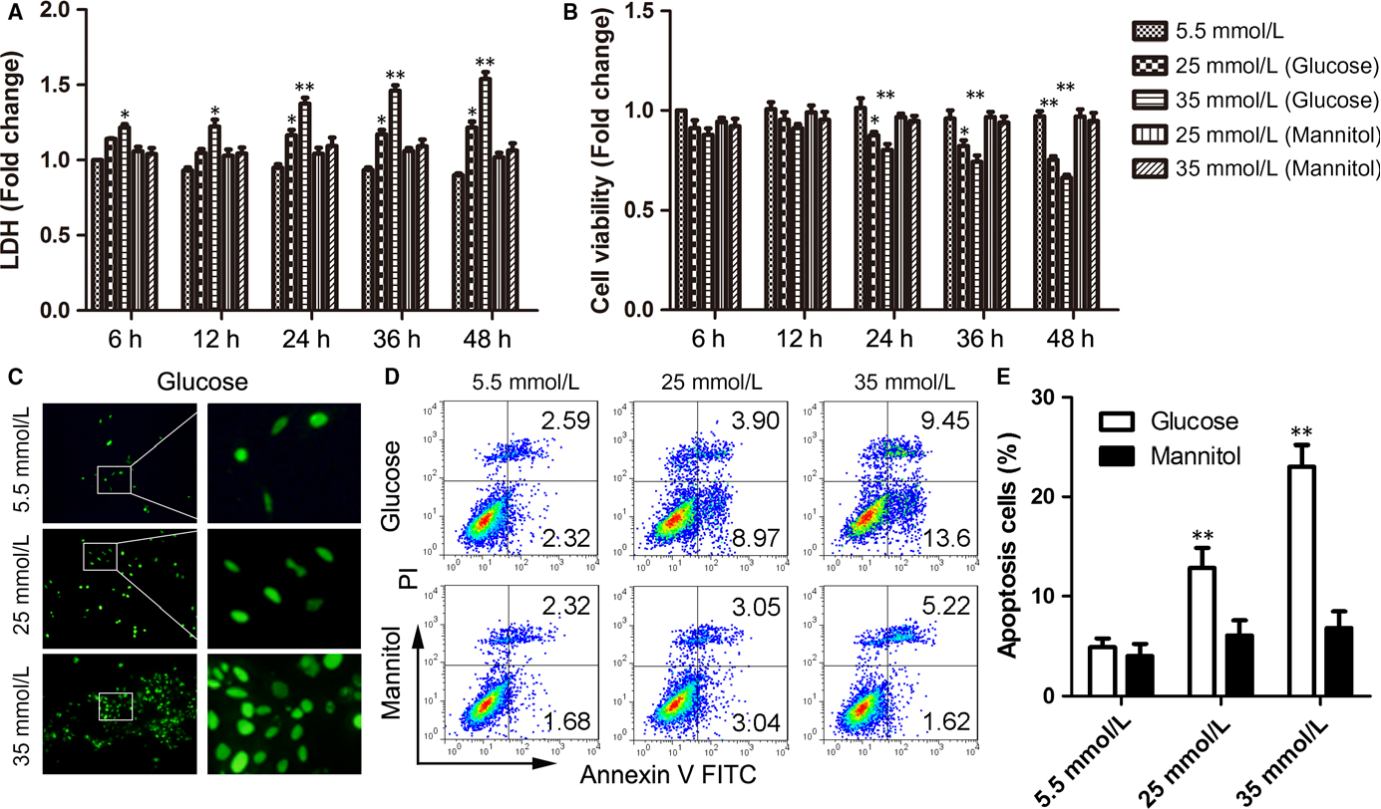
High glucose‐induced H9C2 cell injury and apoptosis. A‐B, LDH (A) and MTS (B) assays were detected in H9C2 cells treated with high glucose for serial hours (6, 12, 24, 36 and 48 h). Mannitol was the control. C, DNA damage was detected through TUNEL in H9C2 cells treated with high glucose for 48 h. D‐E, Representative images of FCM of H9C2 cells treated with high glucose for 48 h (D) and the statistical result of apoptosis cells (E). Data are shown as mean ± SD. *P ≤ .05, **P ≤ .01

To investigate the effect of HG on H9C2 cells, the cells were treated with different concentrations of glucose for 6, 12, 24, 36 and 48 hours. The supernatants were measured using commercial LDH kits, while the residual cells were used for an MTS assay. Although 25 and 35 mmol/L of glucose could induce H9C2 to release more LDH than 5.5 mmol/L of glucose, there are no statistical differences within a short period of time (6 and 12 hours) (Figure 1A). After the H9C2 cells were treated at 24, 36 and 48 hours, we found that HG dramatically resulted in cell damage in a dose‐dependent manner (Figure 1A). The MTS assay also showed that HG reduced myocardial viability in the same manner (Figure 1B). In addition to the H9C2 cells, NRVMs treated with HG simultaneously showed cell damage (Figure S1A,B). Meanwhile, HG signally induced H9C2 cell apoptosis, and this effect was dose‐dependent (Figure 1C‐E). Cell apoptosis in response to HG was confirmed using NRVMs (Figure S1C,D).

To examine whether osmotic pressure affected cardiomyocyte viability, the H9C2 cells were treated with equal concentrations of mannitol. We found that the cell damage and activity were not significantly changed, which suggested that the cell injury depended on glucose itself, not osmotic pressure (Figure 1A,B).

### 3.2 High glucose‐induced cardiocyte injury mainly depended on NLRP3 inflammasome activation

**Figure 2:**
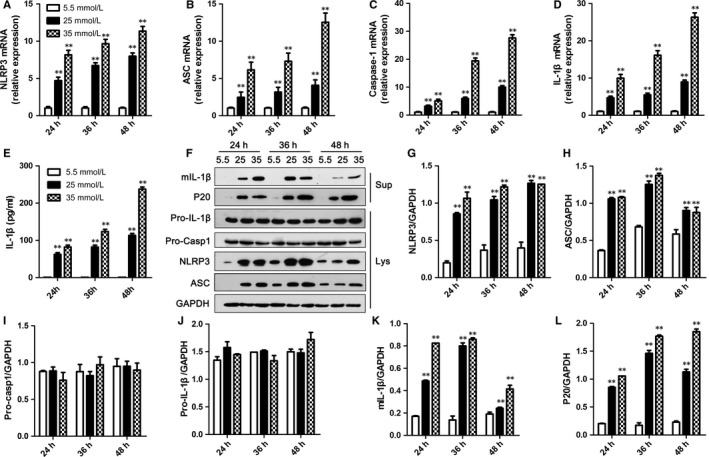
High glucose‐induced NLRP3 inflammasome activation in H9C2 cells. A‐D, The levels of NLRP3 (A), ASC (B), caspase‐1 (C) and IL‐1β (D) mRNAs in H9C2 cells were detected by RT‐PCR and normalized to GAPDH. E, The protein level of IL‐1β in medium supernatants of H9C2 cells was analysed by ELISA. F, Medium supernatants (Sup) and cell lysates (Lys) of H9C2 cells were analysed by immunoblotting as indicated in the text. G‐L, The protein levels of NLRP3 (G), ASC (H), pro‐caspase‐1 (I), pro‐IL‐1β (J), mIL‐1β (K) and P20 (L) were normalized to GAPDH. Data are shown as mean ± SD. **P ≤ .01

To determine the role of NLRP3 inflammasome activation on HG‐induced cardiocyte injury, NLRP3 inflammasome activation in H9C2 and NRVMs was first assessed. HG facilitated the expression of *NLRP3*,* ASC*,* caspase‐1* and *IL‐1*β genes, and this effect was dose‐ and time‐dependent in H9C2 cells and NRVMs (Figures 2A‐D and S2A‐D). We next investigated IL‐1β secretion in H9C2 and NRVMs stimulated with HG through ELISA, which relied on NLRP3 inflammasome activation. As shown in Figures 2E and S2E, HG remarkably stimulated IL‐1β secretion. Meanwhile, the level of IL‐18, another NLRP3 inflammasome‐dependent cytokine, was also signally elevated in NRVMs (Figure S2F).

Moreover, an immunoblotting assay also revealed that NLRP3 and ASC protein levels were elevated in H9C2 cells and NRVMs treated with HG (Figures 2F‐H and S2G), while pro‐caspase‐1 and pro‐IL‐1β protein levels had not changed (Figures 2F,I,J and S2G). Consistent with the ELISA results, HG induced mature IL‐1β production in cell supernatants (Figures 2F,K and S2G). Furthermore, caspase‐1 P20 levels were increased in supernatants of H9C2 and NRVMs stimulated with HG (Figures 2F,L and S2G). Besides these results, HG promoted the expressions of NLRP3, ASC, total caspase‐1 and IL‐1β proteins performed with immunofluorescence (Figure S3).

**Figure 3:**
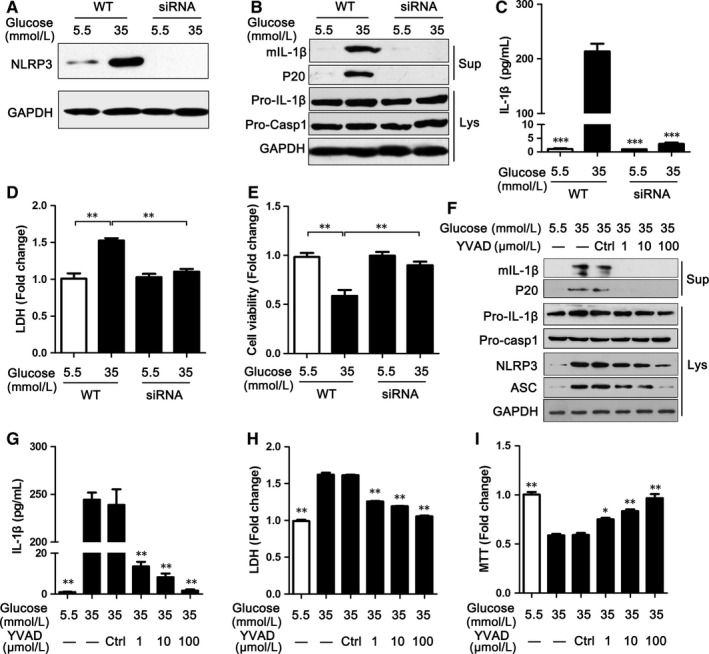
Silencing NLRP3 or inhibiting caspase‐1 suppressed NLRP3 inflammasome activation and improved cardiomyocyte damage. A, The level of NLRP3 protein in H9C2 cells treated with DR5‐targeted siRNA was analysed by immunoblotting as indicated in the text. B, Silencing NLRP3 inhibited caspase‐1 activation and IL‐1β secretion in the medium supernatants (Sup) and cell Lysates (Lys) of H9C2 cells. C, Supernatants of H9C2 cells treated with DR5‐targeted siRNA were analysed by ELISA for IL‐1β. D‐E, LDH (D) and MTS (E) were used to detect cell damage in H9C2 cells treated with siRNA. F, The levels of NLRP3 inflammasome markers were assayed via immunoblotting in H9C2 cells treated with Z‐YVAD‐FMK (YVAD) for 48 h. G, Supernatants of H9C2 cells were also analysed by ELISA for IL‐1β. H‐I, LDH (H) and MTS (I) were used to detect cell damage in H9C2 cells treated with Z‐YVAD‐FMK (YVAD). Data are shown as mean ± SD. *P ≤ .05, **P ≤ .01, ***P ≤ .001

To determine whether HG‐induced NLRP3 inflammasome activation is responsible for cardiocyte injury, the effects of HG on NLRP3 inflammasome activation and cardiocyte injury were assessed after NLRP3 was silenced in H9C2 cells. As shown in Figure 3A, the level of NLRP3 protein was obviously reduced in H9C2 cells treated with siRNA, even under a HG condition. Meanwhile, NLRP3 deficiency blocked HG‐induced NLRP3 inflammasome activation, caspase‐1 p20 production and mature IL‐1β secretion (Figure 3B). Additionally, an ELISA assay for IL‐1β in supernatants showed that IL‐1β diminished in the absence of NLRP3 (Figure 3C). Most of all, the cell injury induced by HG improved when NLRP3 was silenced (Figure 3D,E). Correspondingly, the effects of HG on NLRP3 inflammasome activation and cardiocyte injury were confirmed using the caspase inhibitor Z‐YVAD‐FMK (Figure 3F‐I). Thus, these data indicated that HG‐induced NLRP3 inflammasome activation was responsible for H9C2 cell injury.

### 3.3 High glucose‐induced ROS release accounted for NLRP3 inflammasome activation

**Figure 4:**
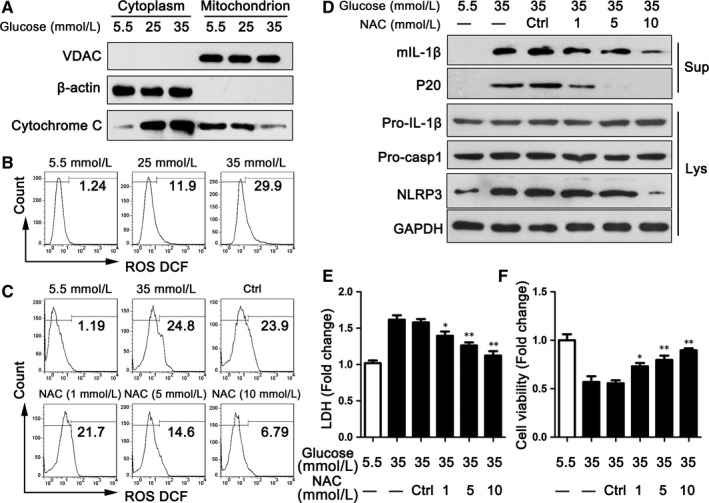
Inhibiting ROS‐suppressed NLRP3 inflammasome activation and improved cardiomyocyte damage. A, The cytoplasm and mitochondrion of H9C2 cells were analysed by immunoblotting for cytochrome *c* after treating with high glucose for 48 h. B, ROS release was detected by FCM in H9C2 cells treated with high glucose for 48 h. C, ROS release was detected by FCM in H9C2 cells treated with high glucose and/or NAC for 48 h. D, The levels of NLRP3 inflammasome markers were assayed via immunoblotting in H9C2 cells treated with NAC for 48 h. E‐F, LDH (E) and MTS (F) were used to detect cell damage in H9C2 cells treated with NAC. Data are shown as mean ± SD. *P ≤ .05, **P ≤ .01

To ascertain the mechanisms of NLRP3 inflammasome activation, we examined ROS release, a common stimulator associated with inflammasome activation. In agreement with previous reports, stimulation was done with HG‐induced cytochrome *c* influx into cytoplasm, which meant the mitochondrion was damaged (Figure 4A). Correspondingly, HG promoted ROS generation, and this increase was dose‐dependent (Figure 4B).

Next, we examined the impact of ROS on NLRP3 inflammasome activation. As shown in Figure 4C, stimulation with NAC reduced the generation of ROS in H9C2, which was also done in a dose‐dependent manner. Additionally, the level of NLRP3 was lower in groups treated with NAC than in the control group (Figure 4D). The expressions of mIL‐1β and cleaved caspase‐1 (P20) were also down‐regulated in NAC‐treated groups than in the DMSO‐treated group (Figure 4D). The cell damage and viability of H9C2 were obviously improved with the pretreatment of NAC compared with DMSO (Figure 4E,F). Thus, these data indicate that ROS release accounted for NLRP3 inflammasome activation induced by HG.

### 3.4 Cytochrome *c* mediated the ROS‐induced NLRP3 inflammasome activation

**Figure 5:**
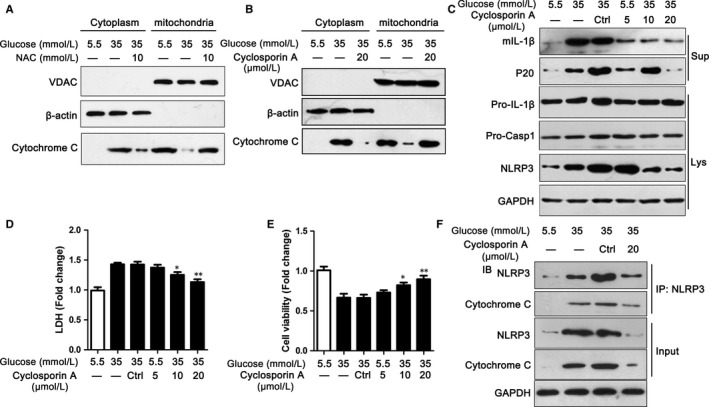
Cytochrome *c* mediated the ROS‐induced NLRP3 inflammasome activation. A, The cytoplasm and mitochondrion of H9C2 cells were analysed by immunoblotting for cytochrome *c* after treating with high glucose and/or NAC for 48 h. B, The cytoplasm and mitochondrion of H9C2 cells were analysed by immunoblotting for cytochrome *c* after being treated with high glucose and/or cyclosporin A for 48 h. C, The levels of NLRP3 inflammasome markers were assayed via immunoblotting in H9C2 cells treated with high glucose and/or cyclosporin A for 48 h. D‐E, LDH (D) and MTS (E) were used to detect cell damage in H9C2 cells treated with cyclosporin A. F, H9C2 cell lysates were analysed by CoIP for NLRP3 and cytochrome *c* as indicated in the text. Data are shown as mean ± SD. *P ≤ .05, **P ≤ .01

Although emerging data showed that ROS can induce NLRP3 inflammasome activation, the exact mechanisms were still uncertain. We next found that NAC not only inhibited ROS release, but also improved cytochrome *c* influx into cytoplasm (Figure 5A). To identify whether cytochrome *c* regulated NLRP3 inflammasome activation, cyclosporine A was used to stimulate H9C2 cells along with HG. Cyclosporine A, as an inhibitor of cytochrome *c*, could effectively inhibit cytochrome *c* influx into cytoplasm, which was also proved by our data (Figure 5B). Furthermore, cyclosporine A could inhibit the expression of NLRP3, which was increased by HG (Figure 5C). The protein levels of mIL‐1β and P20 were also reduced by cyclosporine A (Figure 5D). The cell damage and viability of H9C2 were obviously improved with the pretreatment of cyclosporine A compared with DMSO (Figure 5D,E).

To detect the mechanisms by which cytochrome *c* activated NLRP3 inflammasome, CoIP was used. The data showed that cytochrome *c* could bind to NLRP3, even in the presence of cyclosporine A (Figure 5F). Collectively, these data indicated that cytochrome *c* could bind to NLRP3, which mediated the ROS‐induced NLRP3 inflammasome activation.

### 3.5 *Gps* improved HG‐induced cardiomyocyte damage

**Figure 6:**
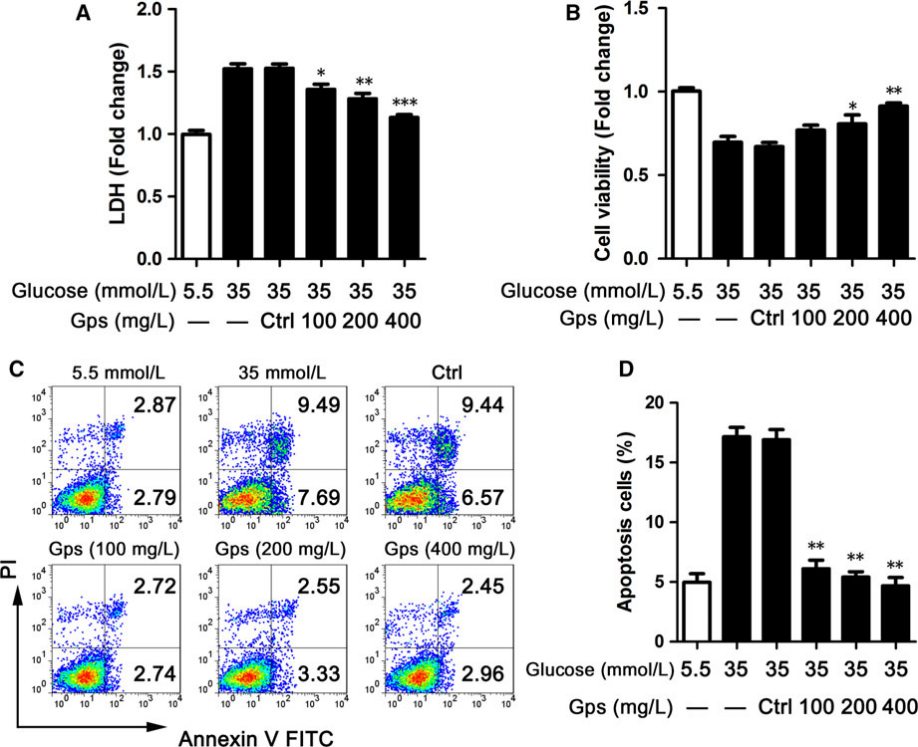
*Gps* improved cardiomyocyte damage. A‐B, LDH (A) and MTS (B) assays were detected in H9C2 cells treated with *Gps* for 48 h. C‐D, Representative images of FCM of H9C2 cells treated with *Gps* for 48 h (C) and the statistical result of apoptosis cells (D). Data are shown as mean ± SD. *P ≤ .05, **P ≤ .01, ***P ≤ .001

To investigate the effect of *Gps* on HG‐induced cardiomyocyte injury, H9C2 cells were treated with *Gps* for 48 hours along with 35 mmol/L glucose. We found that *Gps* noticeably inhibited LDH release and improved cell viability in a dose‐dependent manner (Figure 6A,B). Alternatively, cardiocyte injury could be confirmed by cell apoptosis via FCM. In agreement with Figure 6A,B, HG‐induced cell apoptosis was dose‐dependently inhibited by *Gps* (Figure 6C). The mean percent of apoptosis cells was 17.18% in the HG‐treated group, compared to 5.46%, 5.88% and 5.41% in several concentrations of the *Gps*‐treated groups (Figure 6D).

### 3.6 *Gps* suppressed NLRP3 inflammasome activation by inhibiting ROS release

**Figure 7:**
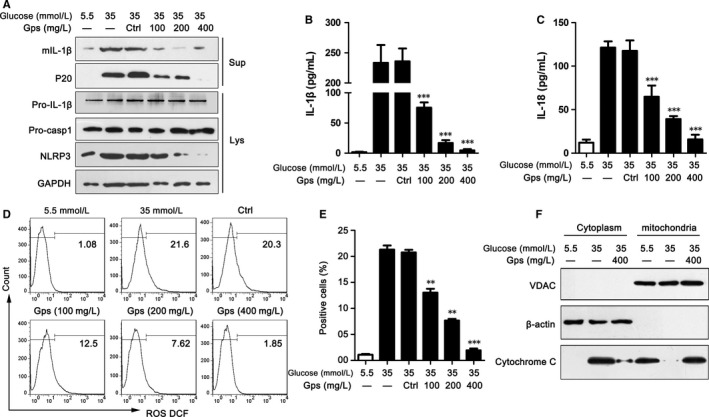
*Gps* suppressed NLRP3 inflammasome activation by inhibiting ROS release. A, The levels of NLRP3 inflammasome markers were assayed via immunoblotting in the medium supernatants (Sup) and lysates (Lys) of H9C2 cells treated with *Gps* for 48 h. B‐C, The levels of IL‐1β (B) and IL‐18 (C) proteins in medium supernatants of H9C2 cells were analysed by ELISA after being treated with *Gps* for 48 h. D‐E, ROS release was detected by FCM in H9C2 cells treated with *Gps* for 48 h (D) and the statistical result of positive cells (E). F, The cytoplasm and mitochondrion of H9C2 cells were analysed by immunoblotting for cytochrome *c* after treating with *Gps* for 48 h. Data are shown as mean ± SD. **P ≤ .01, ***P ≤ .001

To determine whether *Gps*'s improvement of cardiocyte injury relied on regulating NLRP3 inflammasome activation, the effects of *Gps* on NLRP3 inflammasome activation were assessed. *Gps* dramatically inhibited the secretion of IL‐1β and cleaved caspase‐1 (p20), which were induced by 35 mmol/L of glucose (Figure 7A). Meanwhile, the production of NLRP3 was also dose‐dependently inhibited by *Gps* (Figure 7A). Corresponding to the immunoblotting results, the levels of IL‐1β and IL‐18 proteins in medium supernatants of H9C2 cells were dramatically reduced by *Gps* via ELISA (Figure 7B,C). However, in H9C2 cells treated with 5.5 mmol/L of glucose, *Gps* did not influence the production of NLRP3 and ASC (Figure S4A‐C). The secretion of IL‐1β and p20 had also not changed (Figure S4A,D and E). Meanwhile, the ELISA results showed that *Gps* made no difference in the secretion of IL‐1β and IL‐18 (Figure S4F,G).

To determine the mechanisms by which *Gps* inhibited NLRP3 inflammasome activation, we next examined ROS release. As shown in Figures 4B and 7D, stimulation with HG‐induced ROS release in H9C2 cells. Moreover, this effect could be effectively inhibited by *Gps* in a dose‐dependent manner (Figure 7D,E).

Next, we examined the impact of *Gps* on cytochrome *c* influx into cytoplasm. Consistent with the ROS release, *Gps* (400 mg/L) dramatically inhibited cytochrome *c* influx into cytoplasm, which was induced by HG (Figure 7F). Collectively, the above data indicate that *Gps* inhibited ROS release and cytochrome *c* influx into cytoplasm, and then regulated NLRP3 inflammasome activation.

### 3.7 In vivo, gypenosides prevented NLRP3 inflammasome activation and attenuated HG‐induced myocardial injury

**Figure 8:**
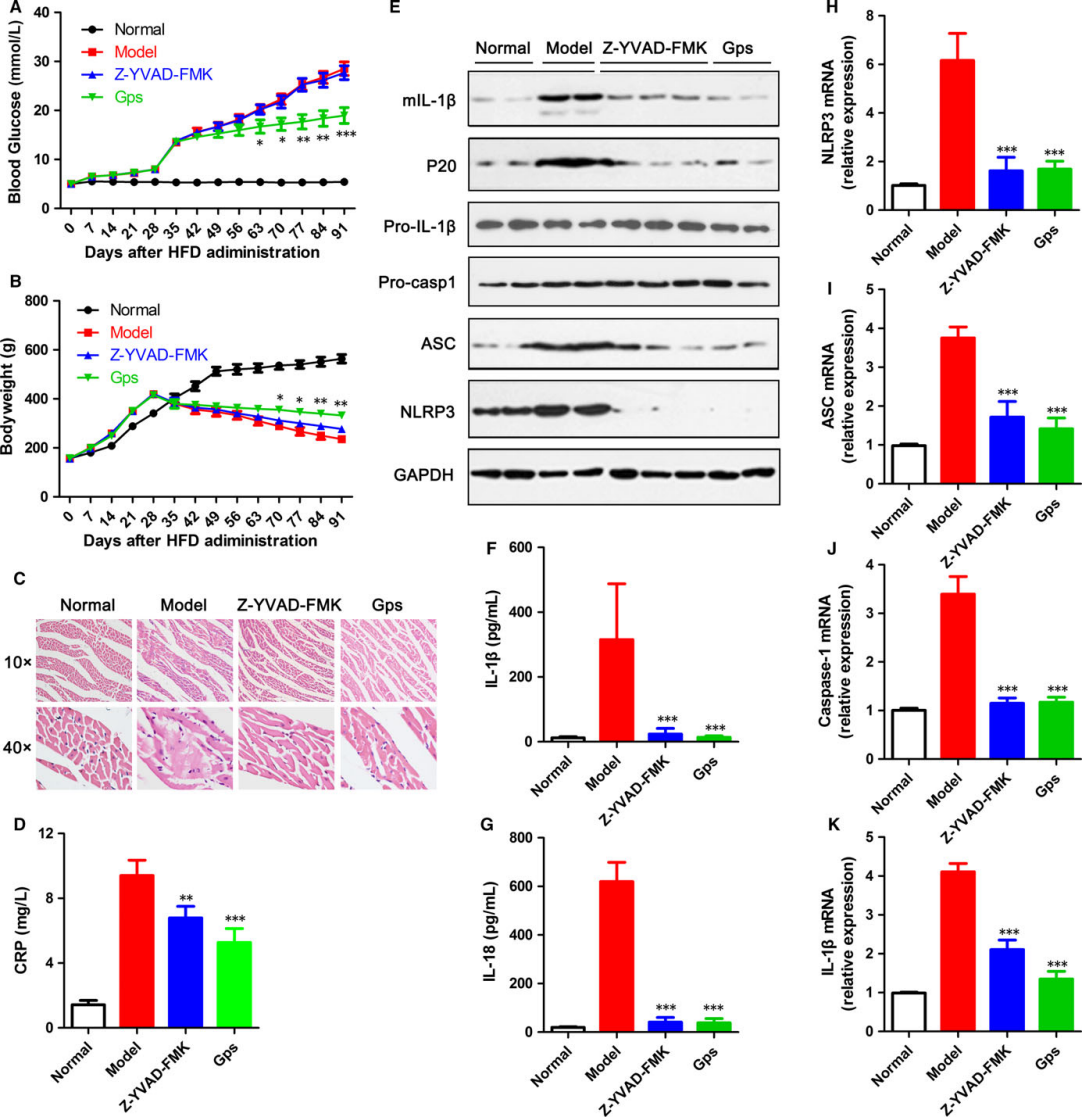
*Gps* inhibited NLRP3 inflammasome activation and DCM development in vivo. A‐B, Blood glucose (A) and body weight (B) were measured every week for 13 wk after HFD administration. C, Myocardial tissue sections were stained with H&E fluid to detect myocardial injury. D, The level of CRP protein in the serum was analysed by ELISA. E, The levels of NLRP3 inflammasome markers were assayed via immunoblotting in myocardial tissue. F‐G, The levels of IL‐1β (F) and IL‐18 (G) proteins in serum were analysed by ELISA. H‐K, The gene levels of NLRP3 (H), ASC (H), caspase‐1 (G) and IL‐1β (K) were assayed via RT‐PCR in myocardial tissue. Data are shown as mean ± SD. *P ≤ .05, **P ≤ .01, ***P ≤ .001

We next investigated the effects of *Gps* in vivo. Mice fed with HFD were treated with *Gps* or Z‐YVAD‐FMK 1 week after i.p. administration of STZ and were monitored for 8 weeks. Corresponding to other research, *Gps* significantly reduced blood glucose and improved the loss of body weight, whereas Z‐YVAD‐FMK had no obvious effects on blood glucose and body weight (Figure 8A,B). Compared to normal mice, mice fed with HFD and then administered with STZ obviously had myocardial injury, whereas the caspase‐1 inhibitor (Z‐YVAD‐FMK) and *Gps* significantly alleviated the induction and injury by HFD (Figure 8C). As an important marker of activation, the level of CRP was markedly higher in mice treated with HFD and STZ than in normal mice. Z‐YVAD‐FMK and *Gps* significantly reduced CRP production (Figure 8D). These data suggest that *Gps* attenuated disease development by reducing blood glucose and inhibiting inflammatory activation in vivo.

To evaluate the role of *Gps* on NLRP3 inflammasome activation in vivo, the total heart protein in these groups was used to detect NLPR3 inflammasome activation. The levels of NLRP3 and ASC were significantly higher in mice treated with HFD and STZ than in normal mice (Figure 8E). Moreover, IL‐1β secretion and P20 production were also dramatically higher (Figure 8E). Z‐YVAD‐FMK and *Gps* significantly reduced NLRP3, ASC and P20 production and IL‐1β secretion (Figure 8E). Consistent with the WB results, *Gps* and Z‐YVAD‐FMK inhibited the production of mature IL‐1β and IL‐18 in the serum of mice (Figure 8F,G). Meanwhile, the mRNA levels of NLRP3, ASC, caspase‐1 and IL‐1β were notably reduced by *Gps* and Z‐YVAD‐FMK (Figure 8H‐K). In a word, *Gps* prevented NLRP3 inflammasome activation in vivo and attenuated HG‐induced myocardial injury.

## 4 DISCUSSION

In this study, we aimed to explore the mechanism of HG‐induced myocardial damage and the effect of *Gps* in HG‐induced myocardial damage. As shown in Figure S5, our work suggests that HG could induce remarkable apoptosis in H9C2 cells, which relied on NLRP3 inflammasome activation and the release of IL‐1β. Moreover, NLRP3 inflammasome activation induced by HG was dependent on mitochondrial cytochrome *c* influx into cytoplasm, which was regulated by ROS. In addition, we observed that *Gps* improved HG‐induced myocardial damage by inhibiting ROS‐dependent NLRP3 inflammasome activation in H9C2 cells and in mice suffering from DCM.

Diabetic cardiomyopathy has become a worldwide health problem, and its primary feature is progressive damage and cardiomyocyte death.^37^ The occurrence of DCM is covert, and the mechanisms remain unclear. Recently, many studies have shown that chronic inflammation and NLRP3 inflammasome activation caused by innate immune cells are important hallmarks of DCM.^14^, ^18^, ^24^ The levels of CCL2, IL‐1β and other proinflammatory cytokines are dramatically reduced in NLRP3‐deficient mice.^38^ Moreover, inhibiting NLRP3 activation or NLRP3 gene silencing exerts a protective role on DCM.^25^, ^39^ Interestingly, some researchers declare that NLRP3 inflammasome activation and subsequent IL‐1β secretion in the heart mainly exist in fibroblasts, endothelial cells and leucocytes, while cardiomyocytes cannot secrete IL‐1β. However, there is considerable evidence that NLRP3 inflammasome activation and IL‐1β secretion induced by HG exist in cardiomyocytes and cause myocardial damage, including in our results.^14^, ^39^, ^40^ In this study, we also found that HG significantly induced NLRP3 inflammasome formation and activation and led to the enhanced production of mature IL‐1β and IL‐18 in H9C2 cells and NRVMs. Meanwhile, NLRP3 gene silencing and/or the caspase‐1 inhibitor (Z‐YVAD‐FMK) dramatically weakened NLRP3 inflammasome activation and the expression of mature IL‐1β not only in H9C2 cells, but also in NRVMs. In vivo, the NLRP3 inflammasome activation was observed in the heart of T2D mice accompanied by DCM. Meanwhile, we found that the level of IL‐1β in serum was dramatically elevated. Interestingly, as one caspase inhibitor, Z‐YVAD‐FMK could not obviously improved the blood glucose and body weight in the mice sustained DCM. However, Z‐YVAD‐FMK could suppressed the activation of caspase 1 and the production of IL‐1β, and had a negative effect on the expression NLRP3 and ASC, which improved the myocardial damage.

Although these data show a close relationship between HG‐induced myocardial damage and NLRP3 inflammasome activation, the mechanism by which HG activates NLRP3 inflammasome is still not clear. Recent studies suggest that mitochondrial ROS is required for NLRP3 inflammasome activation.^41^, ^42^, ^43^ Moreover, many studies have indicated that mitochondrial damage and oxidative stress are important risk factors for DCM and that mitochondrial ROS is a pivotal link between oxidative stress and NLRP3 inflammasome activation.^44^, ^45^ Recently, a study has shown that monocytes and/or MDMs from T2D or DCM patients stimulated with DAMP release an elevated level of ROS.^18^ Meanwhile, ROS inhibitors dramatically inhibit the secretion of mature IL‐1β and IL‐18. Consistent with these data, we found that H9C2 cells stimulated by HG produced elevated levels of ROS, which induced NLRP3 inflammasome activation and subsequent myocardial damage. NAC, a kind of ROS inhibitor, dramatically inhibited NLRP3 inflammasome activation and decreased the secretion of mature IL‐1β.

The NLRP3 inflammasome activation contains two steps.^7^, ^46^ In the first step, which is also called the priming step, PAMP recognizes pathogen response receptors (PRRs) and then activates NF‐κB, which leads to increased levels of *NLRP3*,* IL‐1*β and *IL‐18* genes and proteins. In the second step, many stimuli (uric acid, cardiolipin, dsDNA and so on) induce the formation of the NLRP3 inflammasome and subsequently activate it to produce mature IL‐1β and IL‐18. Initially, ROS were supposed to be one key stimulator participating in the priming step of NLRP3 inflammasome activation, because they were required for NK‐κB activation.^47^ Indeed, we found that the inhibition of ROS by NAC could reduce the production of NLRP3 protein and mature IL‐1β, while the levels of NLRP3, caspase‐1 and IL‐1β did not obviously change (data not shown). This means that ROS may be crucial to the second step of NLRP3 inflammasome activation.

However, the mechanism by which ROS activate the NLRP3 inflammasome is still unclear. Some studies find that ROS can dissociate the thioredoxin‐interacting protein (TXNIP) from the TXNIP‐TXN complex, and free TXNIP induces NLRP3 inflammasome formation and activation.^46^, ^48^ Inhibiting TXNIP can effectively down‐regulate NLRP3 inflammasome activation and IL‐1β secretion.^48^ Moreover, one study has recently shown that ROS can directly induce the formation of the caspase‐1‐ASC complex and activate the NLRP3 inflammasome.^45^ In the current study, we found that ROS could induce cytochrome *c* influx into cytoplasm, which directly bound to NLRP3 and led to NLRP3 inflammasome activation. Cyclosporin A (a cytochrome *c* inhibitor) could significantly inhibit NLRP3 inflammasome activation and mature IL‐1β secretion, while the ROS level was not influenced. Even so, the mechanisms by which ROS regulate cytochrome *c* influx into cytoplasm and cytochrome *c* activates NLRP3 inflammasome deserve further research.

*Gps*, the major component of *Gynostemma pentaphylla* (*Thunb*.) *Makino*, have shown the properties of hypoglycaemic action and anti‐inflammation. In the current study, we found that *Gps* can inhibit NLRP3 inflammasome activation and IL‐1β and IL‐18 secretion, which might result from their ability to reduce the level of mitochondrial ROS. In vivo, *Gps* could reduce the level of blood glucose, inhibit the expression of NLRP3 protein and IL‐1β production and improve myocardial damage. Meanwhile, the generic levels were also reduced in mice administrated with *Gps*, which suggested that *Gps* might regulate the priming step of the NLRP3 inflammasome.

In summary, our data demonstrated that *Gps* inhibited HG‐induced myocardial damage through the ROS‐induced NLRP3 inflammasome activation in vitro and in vivo. Meanwhile, the ROS‐activated NLRP3 inflammasome might result from cytochrome *c* binding to NLRP3. Therefore, *Gps* may be a potential therapeutic solution for inhibiting NLRP3 inflammasome activation and NLRP3‐associated syndromes.

## CONFLICT OF INTEREST

The authors declare no conflict of interests.
