# Supplementary figures and images for: Gypenosides improve diabetic cardiomyopathy by inhibiting ROS‐mediated NLRP3 inflammasome activation
Source: J Cell Mol Med. 2018 Jul 11;22(9):4437–48. doi: 10.1111/jcmm.13743 (PMC6111804; doi:10.1111/jcmm.13743)

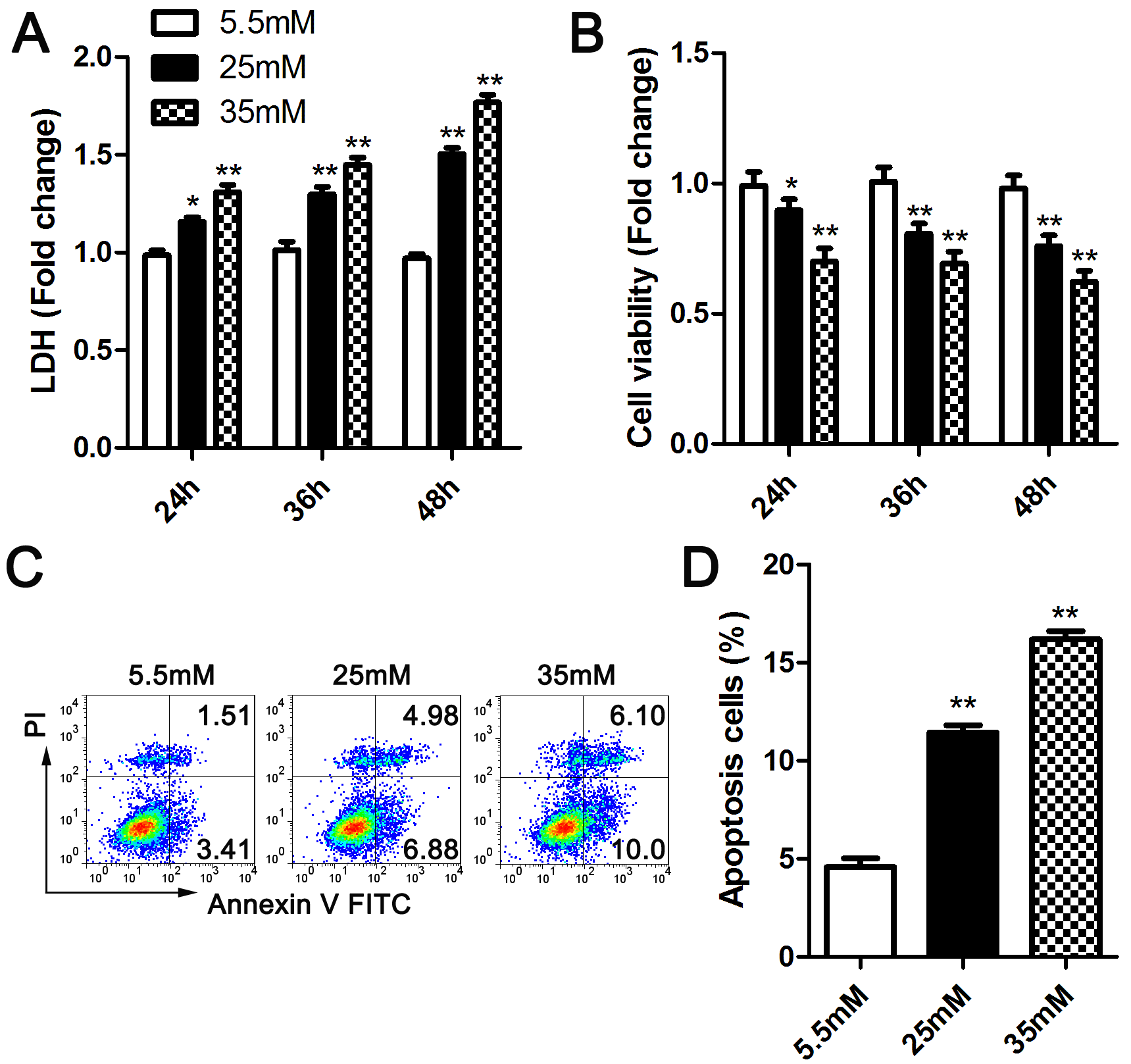

Supplement: Supplementary file 1 [file JCMM-22-4437-s001.tif]

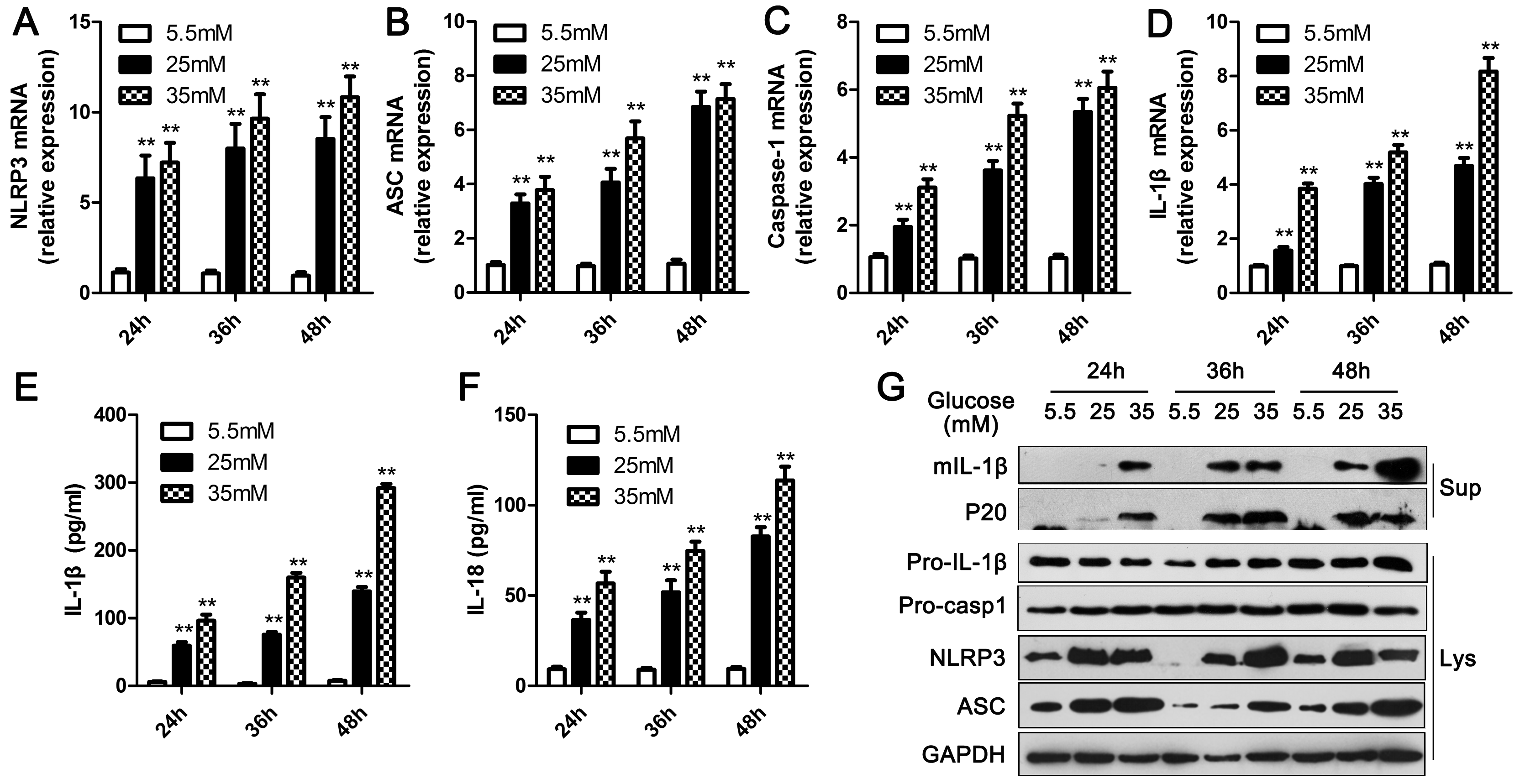

Supplement: Supplementary file 2 [file JCMM-22-4437-s002.tif]

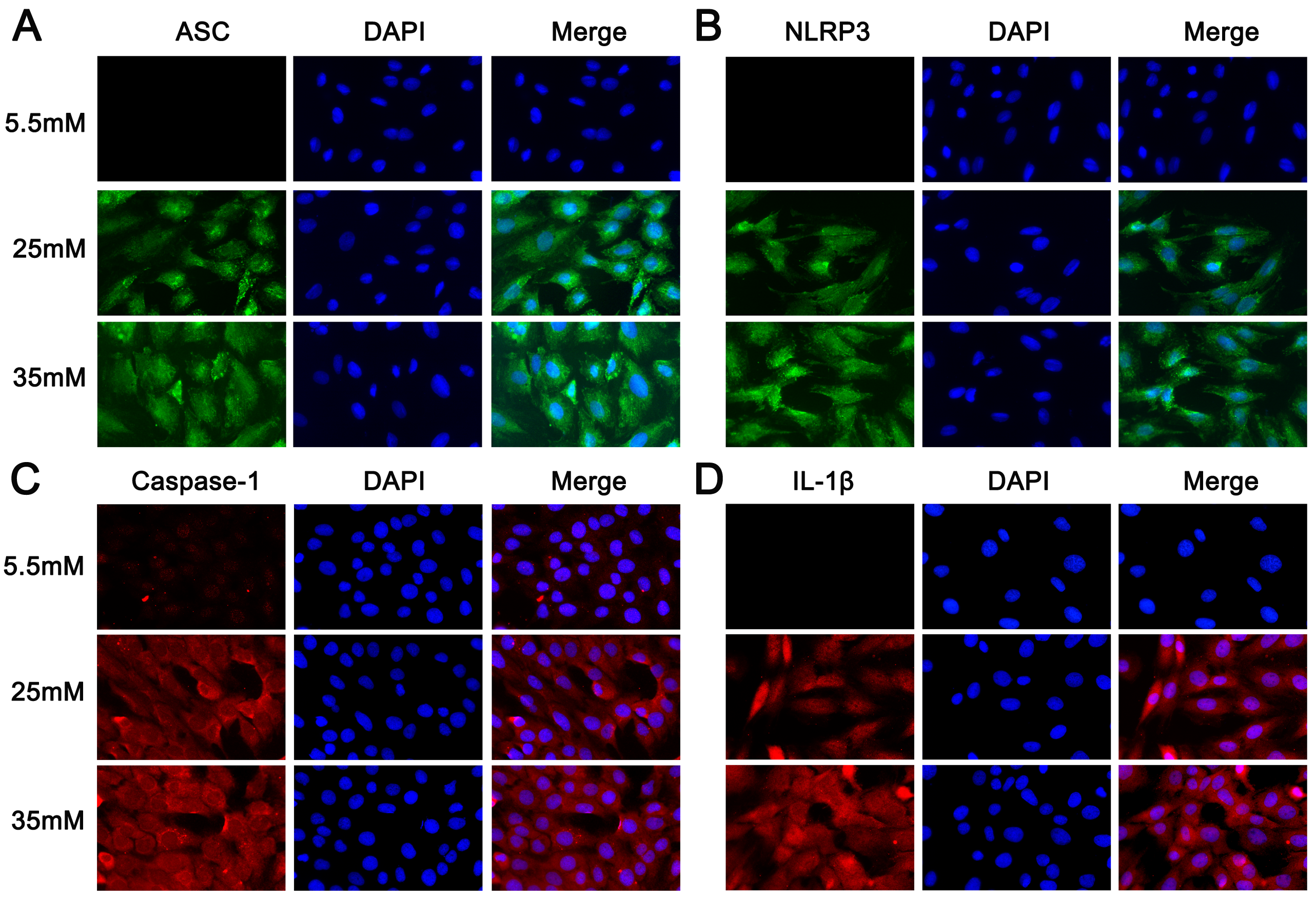

Supplement: Supplementary file 3 [file JCMM-22-4437-s003.tif]

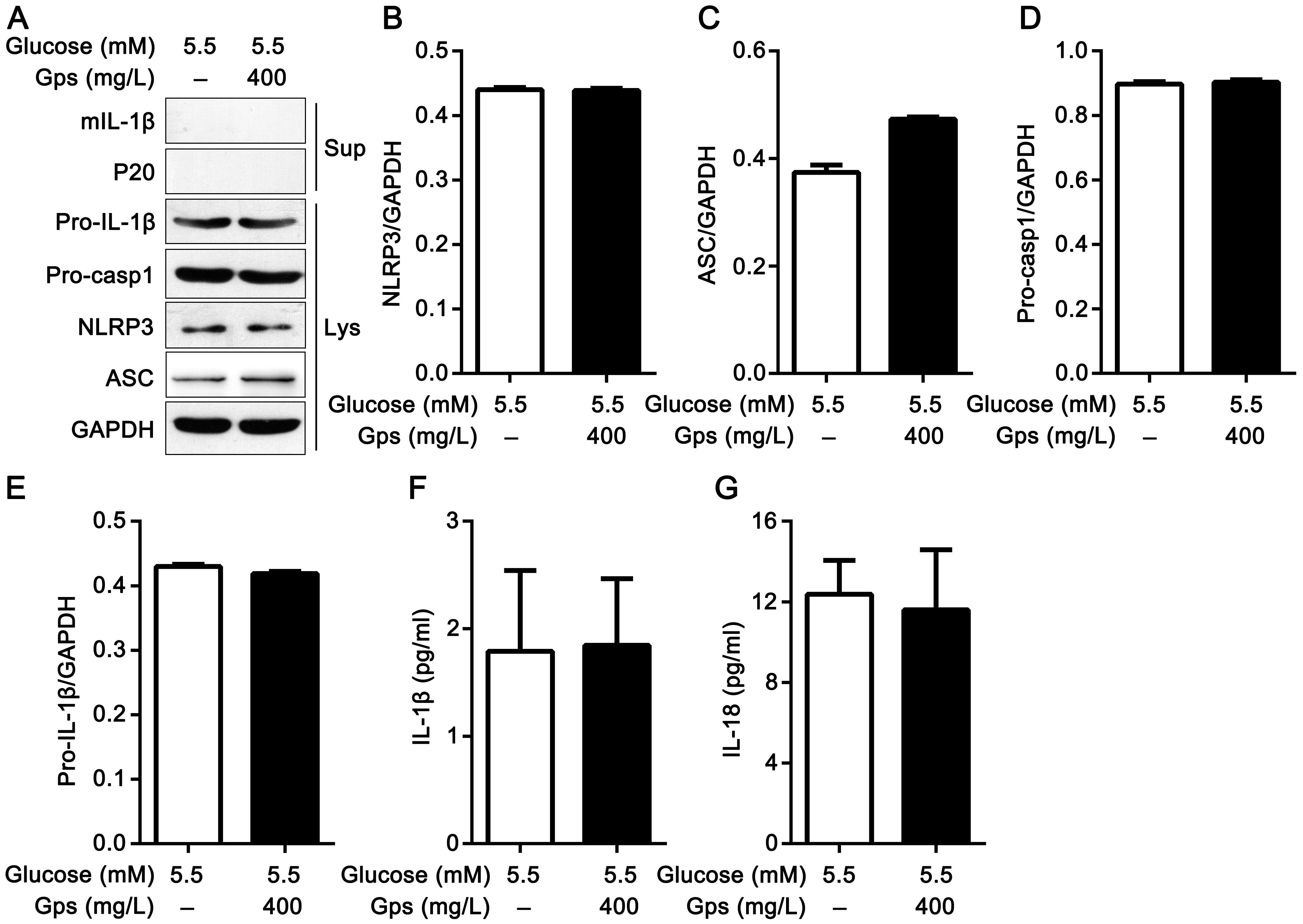

Supplement: Supplementary file 4 [file JCMM-22-4437-s004.tif]

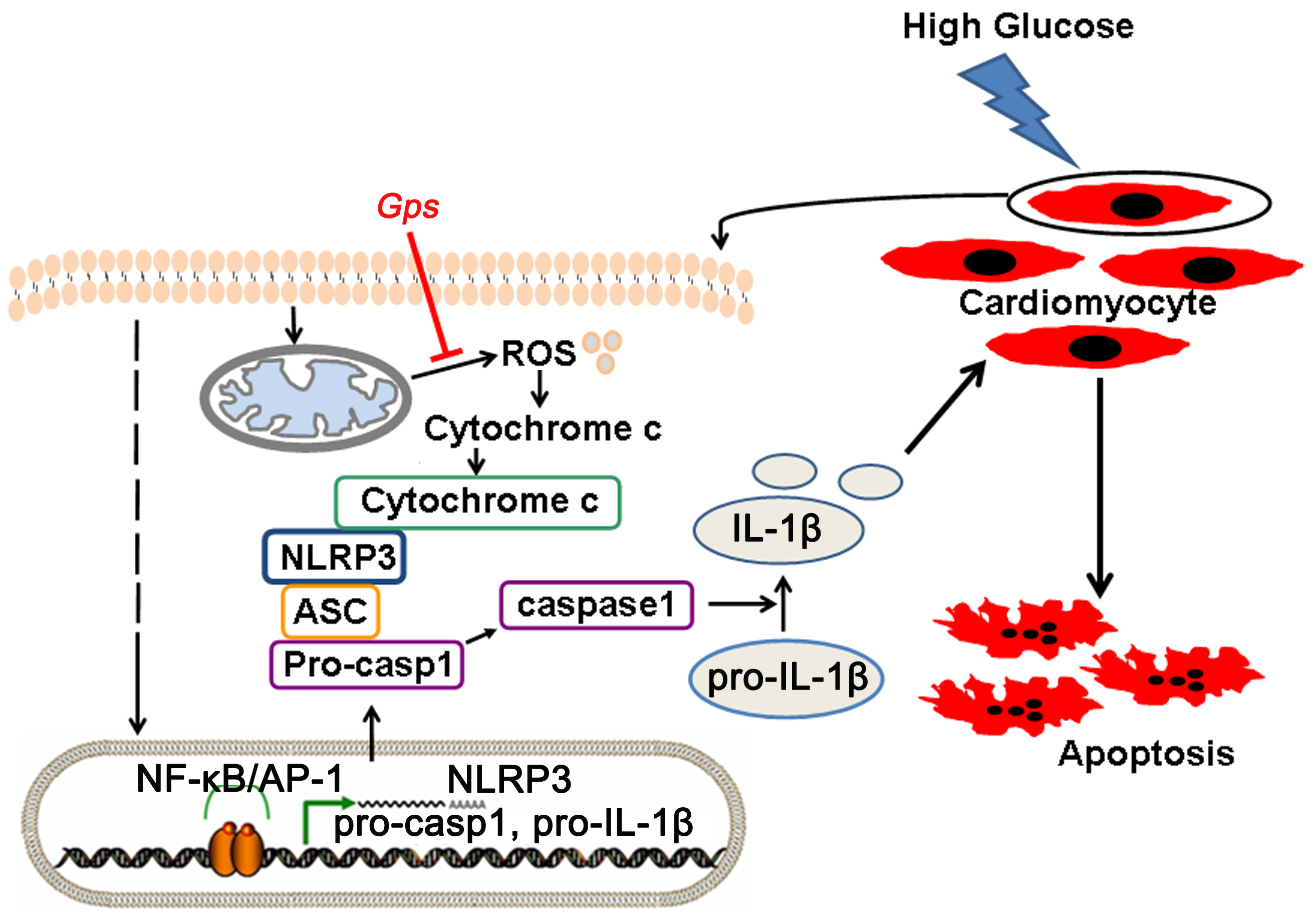

Supplement: Supplementary file 5 [file JCMM-22-4437-s005.tif]
