# Supplementary material for: Gypenosides improve diabetic cardiomyopathy by inhibiting ROS‐mediated NLRP3 inflammasome activation
Source: J Cell Mol Med. 2018 Jul 11;22(9):4437–48. doi: 10.1111/jcmm.13743 (PMC6111804; doi:10.1111/jcmm.13743)
Supplement: Supplementary file 6 [file JCMM-22-4437-s006.docx]

**Table S1.** The primer sequences of GAPDH, NLRP3, ASC, Caspase-1 and IL-1β.

| Gene name | Forward | Reverse |
| --- | --- | --- |
| GAPDH | ACGGGAAACCCATCACCAT | CTCGTGGTTCACACCCATCA |
| NLPR3 | AGCTGCTCTTTGAGCCTGAG | TCTGCTAGGCTCTTTGGTGC |
| ASC | TTGCTGGATGCTCTGTATG | CCAAGTAGGGCTGTGTTTGC |
| Caspase-1 | GACCGAGTGGTTCCCTCAAG | GACGTGTACGAGTGGGTGTT |
| IL-1β | GGCCTCAAGGGGAAGAATC | ATGTCCCGACCATTGCTGTT |
